# Probing the “Dark Matter” of the Human Gut Phageome: Culture Assisted Metagenomics Enables Rapid Discovery and Host-Linking for Novel Bacteriophages

**DOI:** 10.3389/fcimb.2021.616918

**Published:** 2021-03-15

**Authors:** C. Brian Fitzgerald, Andrey N. Shkoporov, Aditya Upadrasta, Ekaterina V. Khokhlova, R. Paul Ross, Colin Hill

**Affiliations:** APC Microbiome Ireland, University College Cork, Cork, Ireland

**Keywords:** bacteriophage, phageome, virome, human gut microbiome, metagenomics, enrichment cultures

## Abstract

Recent years have been marked by the growing interest towards virulent and temperate bacteriophage populations inhabiting the human lower gastrointestinal tract – the gut phageome. A number of studies demonstrated high levels of specificity and temporal stability of individual gut phageomes, as well as their specific alterations in disease cohorts, in parallel with changes in the bacteriome. It has been speculated that phages might have an active role in shaping the taxonomic composition and functional properties of the human gut bacteriome. An overwhelming majority of gut bacteriophages, however, remain uncultured, unclassified, and their specific hosts and infection strategies are still unknown. They are often referred to as “the viral dark matter”. A possible breakthrough in understanding of the phageome can only become possible when a significant proportion of the “the viral dark matter” is identified and linked to bacterial hosts. Here, we describe a method that enables rapid discovery and host-linking of novel bacteriophages in the gut *via* a combination of serial enrichment cultures and shotgun metagenomics of viral DNA. Using this approach dozens of novel and previously known bacteriophages were detected, including the ones infecting difficult-to-culture anaerobic bacteria. The majority of phages failed to produce lysis and propagate on host cultures in traditional assays. The newly identified phages include representatives of *Siphoviridae*, *Myoviridae*, *Podoviridae*, and crAss-like viruses, infecting diverse bacterial taxa of Bacteroidetes, Firmicutes, Actinobacteria, Verrucomicrobia and Proteobacteria phyla. The proposed new method has a potential for high-throughput screening applications for mass discovery of new phages in different environments.

## Introduction

Bacteriophages are present in the human gut in at least the same numbers as bacteria (Shkoporov and Hill, 2019; Sausset et al., 2020). Despite that, the research of the gut phageome dramatically lags behind the rapid progress made in understanding the role of the gut bacteriome in human health and disease. The first attempts to characterise viral communities in human faeces using focussed metagenomic sequencing of virus-like particle (VLP)-enriched filtrates were made a decade ago (Breitbart et al., 2008; Reyes et al., 2010). Since then a considerable body of metagenomic data has been accumulated, showing individual specificity and stability of the phageome (Minot et al., 2013; Manrique et al., 2016; Shkoporov et al., 2019), stepwise progression of the phageome through life (McCann et al., 2018; Liang et al., 2020), in response to dietary changes (Minot et al., 2011) and gastrointestinal pathology (Norman et al., 2015; Clooney et al., 2019). Despite the critical lack of definitive experimental proof, certain evidence from human studies and studies on animal models allow to speculate that the phageome can have an active role in shaping the taxonomic composition and/or influencing the functional properties of the intestinal bacteriome, which eventually affects the physiology of the mammalian host (Ott et al., 2017; Draper et al., 2019; Duan et al., 2019; Hsu et al., 2019).

While the viral metagenomic data from the human gut continues to accumulate rapidly (Ma et al., 2018; Gregory et al., 2020), a critical lack of interpretation of this data becomes more and more obvious. Various metagenomic studies have reported that between 75%–99% of putative viral contigs cannot be taxonomically classified, or linked to any microbial hosts, due to the high degree of sequence divergence with known, previously cultured phages, and absence of a universal taxonomic marker genes (Shkoporov and Hill, 2019). This poorly interpretable fraction of the gut virome has there been nicknamed as ‘the viral dark matter’ (Roux et al., 2015b; Krishnamurthy and Wang, 2017; Clooney et al., 2019).

Large scale isolation, cultivation and cataloguing of phages is therefore required to achieve the same level of phageome interpretation that has already been achieved for the gut bacteriome. Starting with the seminal effort of the Human Microbiome Project, thousands of bacterial strains were cultivated from the human gut and subjected to shotgun genome sequencing, generating a crucial resource of reference genomes against which the metagenomic data can be compared (The Human Microbiome Jumpstart Reference Strains Consortium, 2010; Browne et al., 2016; Zou et al., 2019).

Unlike symbiotic gut bacteria, the majority of which can be propagated in the lab with varied level of effort (Rajilić-Stojanović and de Vos, 2014), isolation and propagation of a bacteriophage requires a suitable bacterial host. Given the narrow host range of many phages (de Jonge et al., 2019), prevalence of temperate phages in the gut (Reyes et al., 2010; Cornuault et al., 2018; Fitzgerald et al., 2018; Cornuault et al., 2020), rapid acquisition of reversible resistance through phase variation of surface receptors and other mechanisms (De Sordi et al, 2019; Hryckowian et al., 2020; Porter et al., 2020), isolation of metagenomically detected, uncultured phages proves to be a daunting task (Dutilh et al., 2014). Traditional approaches of phage isolation, characterisation and linking to specific bacterial hosts rely on detection of plaques formed by phage-mediated lysis of bacterial cells embedded in semi-solid agar. This approach, however, is laborious and depends on many critical elements, such as availability of suitable bacterial host strains, their effective growth in agar media, and the ability of phage to lyse cells efficiently and to diffuse in the agar (Lillehaug, 1997). It appears that many important and highly predominant phage-host pairs, forming the core of the human gut phageome and bacteriome, lack such properties.

In this study, a new rapid, cost-effective and scalable technique for detection and partial characterisation of novel phage from the human gut microbiome is reported. The proposed method uses metagenomic sequencing coupled with culturing to detect specific enrichment (proliferation) of phages. Faecal filtrates obtained from pooled human faecal samples were used as the source of phages in combination with a panel of bacterial host strains typical of the human gut microbiota. The study was carried out in two stages. The first part included enrichment in the presence of various bacterial hosts obtained from culture collections and used two pools of faecal filtrates obtained from healthy volunteers. In the second part, enrichment was performed on bacterial hosts isolated from the same faecal samples that were used as source of phages.

This culture assisted metagenomic approach has yielded dozens of novel phage-host pairs. In fact, it also led to the discovery of φCrAss001, the first ever cultured representative of the crAss-like phages (Shkoporov et al., 2018a). This method has the potential for upscaling in order to perform high-throughput identification and classification of novel phages in the human gut microbiome and other complex microbial ecosystems.

## Method

### Faecal Samples

In order to identify novel human gut phages and simultaneously link them with their hosts we performed phage enrichment experiments with three pooled faecal filtrates. Faecal samples were collected in accordance with study protocol APC055, approved by the Cork Research Ethics Committee. Faecal filtrate pools labelled as “A”, “B”, and “C” were obtained through mixing of 13, 20, and three individual human faecal filtrate samples respectively, diversified with respect to age, gender, and previously established microbiome composition. Collection and storage of faecal samples for pools “A” and “B” was performed essentially as described previously (Shkoporov et al., 2018b). To prepare faecal pool “C”, three volunteers collected their own samples and transported them to the lab where they were processed immediately upon arrival.

Faecal samples from pool “C” were serially diluted and plated on cow rumen fluid-based M2GSC agar (Lopez-Siles et al., 2012), as well as Brain Heart Infusion (BHI) agar supplemented with 0.5% mucin, followed by anaerobic incubation at 37°C for isolation of strict and facultative anaerobes. Bacterial colonies of different morphological types were then streaked out and identified by partial 16S rRNA gene sequencing (Kulagina et al., 2012). In parallel to that, faecal filtrates from individual samples were prepared as follows, 0.5 g of faeces were suspended in 10 ml of cold SM buffer and centrifuged twice, at 5,200×*g* in a swing bucket rotor for 10 min at +4°C. Faecal supernatants were filtered twice using 0.45 µm pore PES membrane filters to remove cells and debris. Finally, pooled faecal viromes “A”, “B”, and “C” were prepared by combining equal volumes of filtrates obtained from individual faecal samples.

### Phage Enrichment

**Figure 1:**
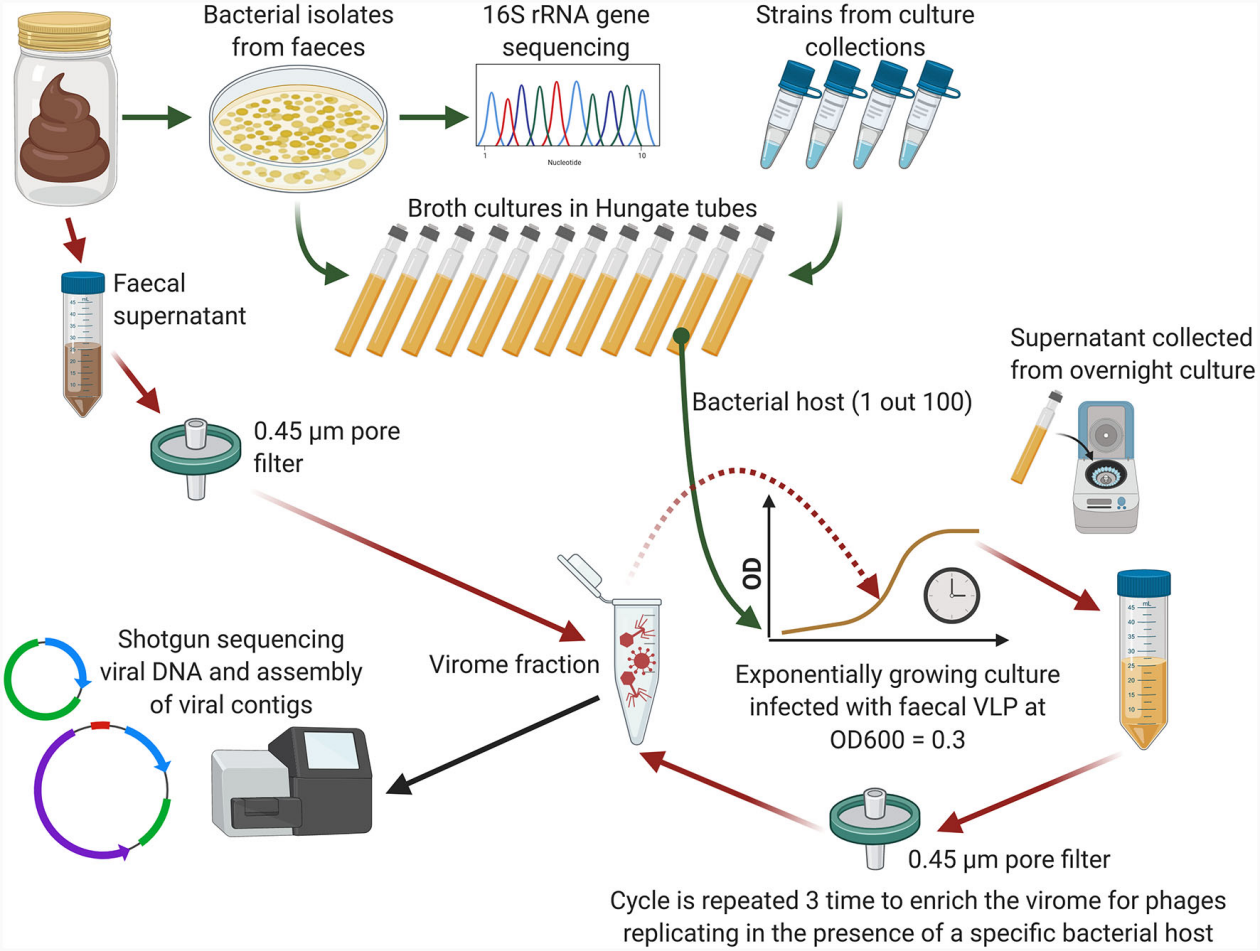
Conceptual diagram of the phage enrichment method (Created using BioRender.com).

A triple-round enrichment of pooled viral filtrates was performed in the presence of exponentially growing pure cultures of 100 different bacterial strains, either sourced from collections or fresh isolates (**Figure 1**). Bacterial strains were selected to represent diverse phylogenetic lineages common to the human gut, including the phyla Bacteroidetes, Firmicutes, Actinobacteria, Proteobacteria, and Verrucomicrobia (**Supplementary Table 1**). For faecal pools “A” and “B”, 26 and 53 bacterial strains were selected, respectively, with partial overlap (n=18) between the strain sets. For faecal pool “C”, 37 strains were isolated from the same three faecal samples used to prepare the pooled viral filtrates.

Bacterial strains were grown anaerobically on yeast extract/casitone/fatty acids (YCFA) broth (Duncan et al., 2002) supplemented with a mixture of carbohydrates (D-glucose, soluble potato starch, D-cellobiose and D-maltose) at concentration of 2 g/L each. Cultures were inoculated into Hungate tubes containing 9 ml of YCFA broth and incubated at 37°C until OD_600 =_ 0.3 was achieved. One millilitre of pooled faecal filtrate (“A”, “B”, or “C”) was then added and incubation was continued at 37°C overnight. Cultures were then centrifuged twice and filtered twice as described above. One millilitre of this filtrate was then added to an exponentially growing culture of the same strain for a second round of enrichment and followed by a third round of enrichment in similar manner. At the end of the third round, viral particles were collected from supernatants using PEG+NaCl precipitation and nucleic acids were extracted as previously described (Shkoporov et al., 2018b). Original faecal filtrate pools before enrichment were processed alongside with the enriched samples.

### Viral DNA Sequencing

Following reverse transcription and multiple displacement amplification [MDA, (Shkoporov et al., 2018b)], shotgun libraries were prepared using Nextera XT DNA Library Preparation Kit (Illumina) and sequenced on an Illumina HiSeq 2500 platform at GATC Biotech AG, Germany. Raw sequencing data is available from NCBI SRA database under BioProject accession PRJNA668036 (**Supplementary Table 1**). Illumina reads were processed, assembled and filtered to remove non-viral contaminating sequences essentially as described elsewhere (Shkoporov et al., 2019). In brief, reads were trimmed and filtered using Trimmomatic v0.36. MetaSPAdes v3.13.0 was used to assemble the reads on a per-sample basis. Contigs (length >1 kb) from all enrichment samples were then pooled together and demultiplexed by picking the longest representatives for each group of contigs with >90% sequence identity and >90% of sequence overlap. Viral contigs were identified using matches to NCBI viral RefSeq database, human gut virome database (Gregory et al., 2020), crAss-like phage genome database (Guerin et al., 2018; Yutin et al., 2020), IMG/VR viral metagenomics database (Paez-Espino et al., 2017) as well as using VirSorter algorithm (Roux et al., 2015a) and presence of three conserved prokaryotic viral protein orthologues (pVOGs) per 10 kb of contig length (Grazziotin et al., 2017). Filtered reads were aligned back to the common demultiplexed database of contigs, to quantify presence of various contigs in the enrichment samples.

## Results

**Figure 2:**
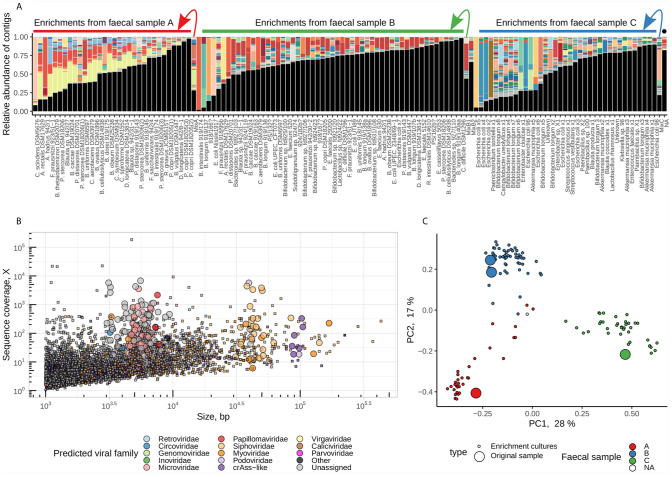
Enrichment of pooled human faecal filtrates in the presence of 100 individual bacterial strains recovers hundreds of viral genomic fragments. **(A)** Compositional bar charts reflecting relative abundance of individual viral genomic contigs (randomly coloured) in enriched and non-enriched original pooled faecal filtrates (located at arrow starts). Samples are grouped by faecal pool used for enrichment (“A”, “B”, or “C”) and ordered by fraction of reads aligned to viral contigs (high to low). Black segments represent fractions of reads aligned to contigs of non-viral origin. Sample labelled with black dot is a blank MDA amplification control. **(B)** distribution of contigs by length and sequence coverage, overlayed by viral taxonomic assignment data. **(C)** PCoA ordination of samples based on Spearman’s rank correlations of relative abundance of viral contigs.

Paired-end short Illumina reads (1.8 ± 1.2M reads per sample, median ± IQR) were assembled and the viral contigs were extracted from the background of contaminating bacterial genomic contigs. After the removal of redundant contigs across 128 samples, a total of 5,553 unique contigs were identified as of potentially viral origin. The fraction of viral contigs per sample varied from as low as 1.6% to as high as 98% of a sequencing space. Samples with high proportion of viral DNA in them tended to consist of one or very few viral contigs suggesting successful enrichment (**Figure 2**). The taxonomic range of recovered viral contigs was very wide and included prokaryotic viruses in the families *Inoviridae*, *Microviridae*, *Siphoviridae*, *Podoviridae*, *Myoviridae*, members of provisional group of crAss-like phages [proposed order Crassvirales (Koonin and Yutin, 2020; Yutin et al., 2020)] as well as different eukaryotic DNA and RNA viruses (**Figure 2**). Viral community composition in enriched viral supernatants reflected the composition of original faecal virome pools, with exception of some samples where successful enrichment was evident. (**Figures 2A**).

**Figure 3:**
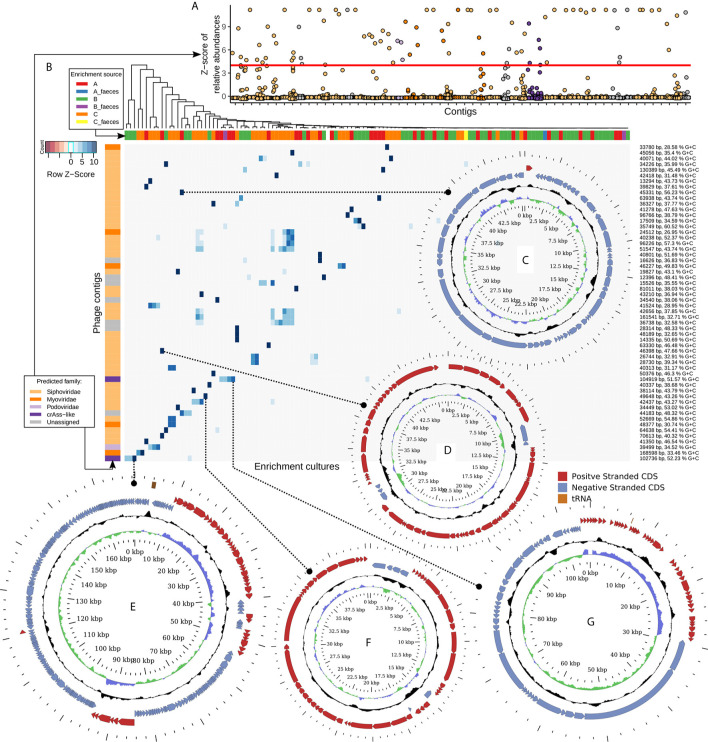
Fifty six strongly enriched viral contigs represent diverse members of the order Caudovirales and replicated in the presence of typical human gut bacteria. **(A)** Z-scores of relative abundance of 56 manually selected viral genomic contigs across all study samples. Specific enrichment in one or a few related strains is a typical result. **(B)** Heatmap of scaled and centred relative abundance of 56 contigs across all 128 samples. **(C)** Circular genome of 35.3 kb *Bacteroides uniformis* DSM6597 phage; **(D)** circular genome of 46.4 kb temperate *Siphoviridae* phage infecting *Anaerostipes hadrus* APC942/1; **(E)** circular genome of a 168.6 kb *E. coli* phage; **(F)** circular genome of a 40.3 kb *Subdoligranulum* sp. APC924/74 inducible prophage; **(G)** circular genome of a 104.9 kb crAss-like phage of *Prevotella stercorea* DSM 18206.

In order to select for viral contigs that were strongly and specifically enriched in bacterial cultures, several steps were performed. Firstly, contigs were taken which had a relative abundance deviating to a Z-score of at least +4 from the mean relative abundance of the same contig over samples prepared from the same viral pool (either “A”, “B”, or “C”, **Figure 3**). Secondly, a contig had to have a relative abundance of at least 1% in any of the enriched samples. Thirdly, a contig had to be either recognized as viral by VirSorter algorithm, or contain at least three pVOGs per 10 kb of its length, or be circular, and have a length of at least 10 kb. The latter step eliminates small circular ssDNA prokaryotic viruses such as *Inoviridae* and *Microviridae*. However, due to the MDA step in our pipeline, which is known to cause strong and stochastic over-representation of circular ssDNA genomes (Roux et al., 2016), we found that these groups produced an especially noisy signal and therefore could not be reliably assigned to any hosts.

The remaining, shortlisted viral contigs of >10 kb (n=89) were manually curated to remove contigs representing fragments of bacterial genomes with prophages in them, as well as contigs enriched ambiguously between different bacterial taxa. A final set of 56 specifically enriched contigs (**Figure 3**, **Supplementary Table 2**) represented complete or nearly complete phage genomes assigned to viral families *Siphoviridae* (n=41, enriched in a broad range of hosts), *Myoviridae* (n=5, enriched in *Escherichia coli*, *Klebsiella* sp., *Akkermansia muciniphila*, *Bifidobacterium longum*), *Podoviridae* (n=1, *E. coli*), and crAss-like phages (n=2, *Bacteroides intestinalis* and *Prevotella stercorea*).

Some select examples of the identified phages include complete circular genomes of (i), a 45.3 kb *Bacteroides uniformis* DSM6597 phage (*Siphoviridae*, **Figure 3**) previously observed in human gut metagenomes; (ii), a 46.4 kb temperate *Siphoviridae* phage infecting *Anaerostipes hadrus* APC942/1 (**Figure 3**); (iii), a 168.6 kb *E. coli* phage (**Figure 3**), 97% identical to previously isolated phage vB_EcoM-fHoEco02 [genus *T4virus*, family *Myoviridae*, (Kiljunen et al, 2018)]; (iv), a 40.3 kb *Siphoviridae* prophage spontaneously inducing from the chromosome of *Subdoligranulum* sp. APC924/74 [**Figure 3**; (Fitzgerald et al., 2018)]; and (v), a 104.9 kb crAss-like phage (**Figure 3**), belonging to subfamily *Deltacrassvirinae*, apparently using a non-standard genetic code [suppression of UAG codon is required to translate most of the genes (Guerin et al., 2018; Yutin et al., 2020)] and enriching in the presence of *Prevotella stercorea* DSM 18206. Another crAss-like phage replicating on *Bacteroides intestinalis* APC919/174 and identified as part of this study was previously reported (Shkoporov et al., 2018a).

**Figure 4:**
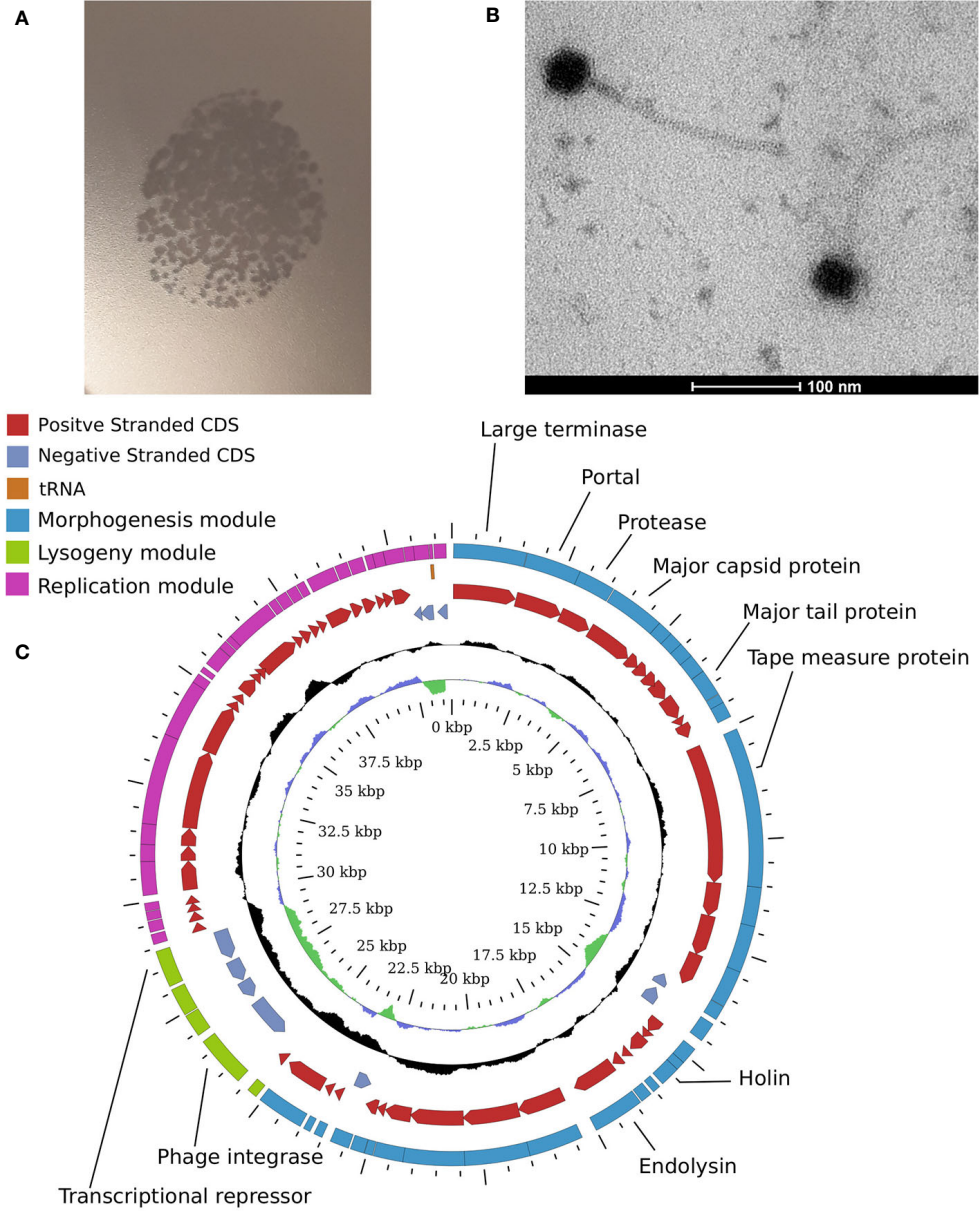
Temperate Siphoviridae phage φCs5676-1 isolated from one of the enriched filtrates infects *Clostridium scindens* DSM 5676. **(A)** formation of plaques in 0.4% YCFA-GSCM agar overlays with *C*. *scindens* DSM 5676; **(B)** transmission electron micrograph of φCs5676-1 particles at 160,000× magnification; **(C)** circularised map of φCs5676-1 genome showing distinct morphogenesis, lysogeny, and replication modules.

In order to confirm the enrichment results we attempted to isolate some of the detected phages in pure cultures using plaque formation assays with viral supernatants obtained from *Anaerostipes hadrus* APC942/1, *Bacteroides uniformis* DSM6597, *Prevotella stercorea* DSM 18206, and *Clostridium scindens* DSM 5676 in addition to previously reported *Bacteroides intestinalis* APC919/174. Overlays of the same strains were used as indicator cultures. Despite being enriched in the supernatants, most phages except for φCs5676-1 (infects *Clostridium scindens* DSM 5676, **Figure 4**) and φcrAss001 (*Bacteroides intestinalis* APC919/174) failed to produce visible lysis in spot and plaque formation assays. This is hardly surprising, as some previous studies have already shown that many naturally occurring gut bacteriophages are difficult to isolate using traditional culturing methods (Dutilh et al., 2014; Porter et al., 2020).

Phage φCs5676-1 has displayed a siphovirus-like morphology (**Figure 4**) and was able to propagate in liquid cultures of *Clostridium scindens* DSM 5676 to levels of 1.2×10^9^ pfu/ml. The 41.3 kb genome of this phage is linear and contains distinct morphogenesis, lysogeny and replication modules typical for temperate *Siphoviridae* phages. To the best of our knowledge, phage φCs5676-1 is the first cultured virus infecting *Clostridium scindens* and can represent a valuable tool for future studies with this common human gut symbiont.

## Discussion

A growing body of metagenomic studies continue to deliver new data on the human phageome composition and its changes in various physiological and pathological states. However, any meaningful interpretation of phage differential abundance will never become possible with the current scarcity of knowledge on biodiversity, phylogeny, infection modes and host ranges of the huge majority of gut-associated phage species. Large scale efforts are required in order to culture and catalogue microbiome phages, much similar to previous efforts of building collections and genome databases of reference bacterial strains for human microbiome research (The Human Microbiome Jumpstart Reference Strains Consortium, 2010).

Here we propose an enrichment- and metagenomic sequencing-based method for rapid discovery and host-assignment of novel phages. A similar method for novel phage discovery and host-linking, which relies on binding to isolated host envelopes, has recently been proposed (de Jonge et al., 2020). Despite its clear advantages the competing method suffers from a number of limitations, most importantly, its inability to distinguish between adsorption and true ability of a given phage to infect a particular host strain. By contrast, our approach results in detection of phages which are genuinely amplified in the presence of host strain. At the same time, this method does not completely rely on the phage ability to produce lysis zone in agar overlays. Furthermore, this method can be applied to large panels of bacterial indicator strains and pooled human faecal samples (or other clinical or environmental samples) to discover phages without prior knowledge of host specificity or infection modes. It is noteworthy that the method is not only capable of detecting the replication of virulent and temperate bacteriophages, exogenous to the indicator strains used, but also induction of previously known and unknown prophages, as demonstrated by detection of circularised *Subdoligranulum* sp. APC924/74 prophage genome and some other complete or nearly complete prophage sequences (**Supplementary Table 2**).

For demonstration purposes, in this study we selected only 56 strong phage candidates out of hundreds of potentially enriched sequences with less certain identification, and/or fragmented genome assemblies. Sequences that were left behind would potentially require more enrichment cycles to become dominant in VLP fraction and/or more bioinformatic efforts for complete genome assembly. Only one out of 56 is represented in NCBI viral RefSeq database and eight were previously sequenced as prophages in genomes of published gut bacterial isolates (**Supplementary Table 2**). Two out of the 56 genomes belong to the provisional group of crAss-like phages, highly abundant in the human gut and associated with the phylum Bacteroidetes. One of the detected genomes belongs to the phage which infects *P. stercorea* and apparently uses a non-standard genetic code with suppression of UAG stop codon throughout its genome (Yutin et al., 2020). This phage belongs to a previously proposed genus VIII of subfamily *Deltacrassvirinae* of crAss-like phages, a subgroup which is highly abundant in non-Western human populations with high faecal *Prevotella* counts (Gorvitovskaia et al., 2016; Guerin et al., 2018). Of note, only two highly enriched phages could subsequently be isolated from culture supernatants using traditional plaque-formation assays. This can be indicative of inability of many human gut phages to reproduce visible lysis in cultures off their host strains and further reinforces the need of alternative strategies for phage detection, identification and characterization, such as the method described here.

To maximize the chances of detecting cognate phage-host pairs, enrichments in the experimental rounds (faecal pool ‘C’) were conducted with bacterial strains isolated from the same faecal sample. Interestingly, 34 out 56 strongly enriched, strongly phage-like sequences in this work resulted from this part of the study, despite it being carried out with only 37 out of 100 used bacterial strains and only 3 faecal samples, compared to 13 and 20 used in faecal pools “A” and “B”, respectively. This result suggests that isolation of phage host-pairs from the same faecal sample can potentially be a much more productive approach compared to screening for phage in faecal samples, using unrelated indicator strains of bacterial hosts.

In conclusion, we propose that this method or its modifications can be used in a large scale future efforts for mass discovery, identification and cataloguing of phages in host-associated and environmental microbiomes. The method can be potentially adapted to use with microtitre plates with the aid of liquid-handling robots for high-throughput screenings. Given a potentially crucial role of phages in shaping the bacterial portion of gut microbiome, improved phage databases will undoubtedly constitute a key resource towards the new level of microbiome interpretation in health and disease and future microbiome manipulation strategies.

## Data Availability Statement

Nucleotide sequence data presented in this study can be found in NCBI databases under BioProject accession PRJNA668036.

## Ethics Statement

The studies involving human participants were reviewed and approved by Cork Research Ethics Committee. The patients/participants provided their written informed consent to participate in this study.

## Author Contributions

CF, AS, and AU conceived and performed the study. EK assisted in phage isolation and propagation and prepared samples for electron microscopy. RPR and CH secured the funding, provided guidance and advice. All authors contributed to the article and approved the submitted version.

## Funding

This research was conducted with the financial support of Science Foundation Ireland (SFI) under Grant Number SFI/12/RC/2273, a Science Foundation Ireland’s Spokes Programme which is co-funded under the European Regional Development Fund under Grant Number SFI/14/SP APC/B3032, and a research grant from Janssen Biotech, Inc. The funder was not involved in the study design, collection, analysis, interpretation of data, the writing of this article or the decision to submit it for publication.

## Conflict of Interest

The authors declare that the research was conducted in the absence of any commercial or financial relationships that could be construed as a potential conflict of interest.
